# Light and circadian regulation of clock components aids flexible responses to environmental signals

**DOI:** 10.1111/nph.12853

**Published:** 2014-05-20

**Authors:** Laura E Dixon, Sarah K Hodge, Gerben van Ooijen, Carl Troein, Ozgur E Akman, Andrew J Millar

**Affiliations:** 1 SynthSys, University of EdinburghKings Buildings, Mayfield Road, Edinburgh, EH9 3JD, UK; 2 Department of Crop Genetics, John Innes CentreNorwich Research Park, Norwich, NR4 7UH, UK; 3 Department of Astronomy and Theoretical Physics, Lund University223 62, Lund, Sweden; 4 Centre for Systems, Dynamics and Control, College of Engineering, Mathematics & Physical Sciences, University of ExeterExeter, EX4 4QF, UK

**Keywords:** biological clocks, flexibility, marine algae, mathematical analysis, nonlinear dynamics, photoperiod, systems biology

## Abstract

- The circadian clock measures time across a 24 h period, increasing fitness by phasing biological processes to the most appropriate time of day. The interlocking feedback loop mechanism of the clock is conserved across species; however, the number of loops varies. Mathematical and computational analyses have suggested that loop complexity affects the overall flexibility of the oscillator, including its responses to entrainment signals.
- We used a discriminating experimental assay, at the transition between different photoperiods, in order to test this proposal in a minimal circadian network (in *Ostreococcus tauri*) and a more complex network (in *Arabidopsis thaliana*).
- Transcriptional and translational reporters in *O. tauri* primarily tracked dawn or dusk, whereas in *A. thaliana*, a wider range of responses were observed, consistent with its more flexible clock. Model analysis supported the requirement for this diversity of responses among the components of the more complex network. However, these and earlier data showed that the *O. tauri* network retains surprising flexibility, despite its simple circuit.
- We found that models constructed from experimental data can show flexibility either from multiple loops and/or from multiple light inputs. Our results suggest that *O. tauri* has adopted the latter strategy, possibly as a consequence of genomic reduction.

## Introduction

Circadian rhythms are biological oscillations of *c*. 24 h and can be identified through their persistence under constant environmental conditions (Harmer, 2009). For an oscillator to be physiologically useful, it must be able to synchronize (entrain) to local environmental conditions. Without entrainment, the timing information provided by the innate circadian mechanism would offer little biological relevance to the organism. However, once entrained, the circadian oscillator is able to regulate the phase of a variety of biological processes to coincide with daily and seasonal cycles (Daan, 1998). These processes include gene regulation, protein stability and metabolic regulation, and act to control a diverse range of mechanisms such as plant growth, abiotic and biotic responses and flowering (Harmer, 2009). Therefore, an entrained circadian oscillator increases the fitness of an organism (Dodd *et al.*, 2005; Harmer, 2009).

Although the core components of circadian oscillators differ between species, the network topology appears universal, being composed of interlocking negative and positive feedback loops (Young & Kay, 2001; Harmer, 2009). As a single negative feedback loop is sufficient to yield a viable entrained oscillator, several theoretical and experimental studies have tested what advantage might be conferred by the more complex multiloop architectures that are observed in many of the model species (Harmer, 2009).

A key prediction that has emerged from this work is that multiple loops increase a clock network's global flexibility, as quantified by its flexibility dimension – a quantitative measure of the degree to which biochemical perturbations can modify the clock dynamics. Clocks that have high flexibility are expected to possess an enhanced capacity to respond to different environmental inputs at different times (Rand *et al.*, 2006; Rand, 2008; Akman *et al.*, 2010; Edwards *et al.*, 2010). The calculations of global flexibility used in these studies have been based on mathematical clock models that are closely constrained by experimental data, as these enable the full set of possible dynamic behaviours that can be generated by a particular network structure to be explored. Unfortunately, direct confirmation of these predictions through experimental measurements is impracticable, because of the exponentially large number of *in vivo* parameter perturbations that would be required. Consequently, previous work has examined entrainment behaviour under different environmental conditions to test particular aspects of global flexibility, such as its influence on circadian phase homeostasis (Akman *et al.*, 2008, 2010; Edwards *et al.*, 2010). One particular manifestation of high global flexibility appears to be an enhanced capacity for entrained phase to be tuned by light and temperature signals (Akman *et al.*, 2008, 2010; Edwards *et al.*, 2010). In Akman *et al*. (2010), a general measure of the responsiveness of an entrained circadian rhythm to light : dark transitions was introduced, the dusk sensitivity. By applying this measure to a range of clock models, it was predicted that high global flexibility results in a broader set of individual clock components exhibiting flexible phase responses under photoperiod changes (Akman *et al.*, 2010; Edwards *et al.*, 2010). Such flexibility was observed in *Arabidopsis thaliana* plants under different photoperiods (Millar & Kay, 1996; Edwards *et al.*, 2010) and non-24 h cycles (Dodd *et al.*, 2014), as well as in the fungus *Neurospora crassa* (Akman *et al.*, 2010 and references therein).

*Arabidopsis thaliana* is a model higher plant species in which circadian rhythms have been extensively studied (Harmer, 2009). Experimental data have enabled a quantitative understanding of the core circadian signalling network, and this has been supported through the development of mathematical models (reviewed in Bujdoso & Davis, 2013). The first such model (L2005A) was based on a single transcription/translation feedback loop (Locke *et al.*, 2005a). This loop is between a pseudoresponse regulator *TIMING OF CAB 1* (*TOC1*) and the MYB-related transcription factors *CIRCADIAN CLOCK ASSOCIATED 1* (*CCA1*) and *LATE ELONGATED HYPOCOTYL* (*LHY*; Alabadi *et al.*, 2001). Subsequent models have expanded this feedback loop to include additional proteins and regulation (L2005B; Locke *et al.*, 2005b), such as the observed morning regulation via *PSEUDO RESPONSE REGULATOR* proteins PRR7 and PRR9 (L2006; Locke *et al.*, 2006; Zeilinger *et al.*, 2006), and later PRR5 (P2010; Pokhilko *et al.*, 2010). The models predicted functional interactions between components that would be required to account for the circadian regulation observed experimentally, such as the X and Y genes proposed in L2005B (Locke *et al.*, 2005b), and helped to identify candidate molecules, such as the repressor proteins PRR5 and TOC1 (Pokhilko *et al.*, 2010, 2012). In particular, the observed repressive regulation by the Evening Complex – comprising EARLY FLOWERING 3 and 4 (ELF3, ELF4) and LUX ARRYTHMO (LUX) proteins – replaced the hypothetical component Y (P2011; Pokhilko *et al.*, 2012). Thus, the *A. thaliana* model has seen a dramatic increase in the number of loops and light inputs comprising the network (see Supporting Information, Fig. S1a,d–g). To determine the function of these loops, it was necessary to experimentally and mathematically test a comparable system with different loop complexity.

The isolation and sequencing of a microalga, *Ostreococcus tauri*, has allowed its circadian network to be experimentally characterized (Derelle *et al.*, 2006; Corellou *et al.*, 2009). At the level of transcription/translation, the *O. tauri* clock may only contain one orthologue of the *A. thaliana CCA1* and one of *TOC1* (Corellou *et al.*, 2009). This minimal, single-loop mechanism, shown schematically in Fig. S1(b), has also been mathematically modelled with biochemical detail similar to the *A. thaliana* models (T2011; Troein *et al.*, 2011) and in simpler models (Thommen *et al.*, 2010). Reporter data are equally consistent with the core oscillator, having a single feedback loop with three inhibitory components, an architecture that is still far simpler than the *A. thaliana* network (Ocone *et al.*, 2013).

Both the *A. thaliana* and *O. tauri* models capture a wide range of the available experimental data, and so the topologies of these models can be considered as fair approximations to the corresponding biological systems. Utilizing the mathematical models, a more detailed investigation into how circadian networks respond to changing entrainment can be made, as all modelled system components can be simultaneously examined. Given the proposed correlation between loop complexity and flexibility, it would be anticipated *a priori* that a single-loop architecture (*O. tauri*) will respond differently to changing photoperiods compared with a multiloop system (*A. thaliana*). Interestingly, preliminary studies in *O. tauri* have demonstrated that its simple, single-loop architecture yields high flexibility of the entrained phase under different photoperiods (Troein *et al.*, 2011), both in the model and in experiments where phase was assessed after a transition into constant light, equivalent to the studies of dusk sensitivity in *A. thaliana*. Such experiments assess entrainment under a range of conditions, but not the ability of the clock to re-entrain during a change from one entraining condition to another.

This study aimed to address a key question that arose from these observations: how do acute light regulation and loop number influence re-entrainment? To address this question, the phase of circadian networks in *A. thaliana* and *O. tauri* was measured during transitions between stable photoperiods. These networks comprise similar proteins but differ in the total number of constituent feedback loops. Mathematically comparing the entrained and free-running patterns generated by oscillators with different numbers of feedback loops and light inputs enabled a further aspect of global flexibility to be empirically tested, providing insights into the different biochemical mechanisms through which flexible circadian responses can be achieved.

## Materials and Methods

### *A. thaliana* growth conditions

The luciferase (LUC) reporter lines *CCA1*::*LUC*,*TOC1*::*LUC* and *GI*::*LUC* have been previously described (Edwards *et al.*, 2010; Pokhilko *et al.*, 2010). The seeds were sterilized in 10% commercial bleach solution and stratified for 4 d at 4°C. Unless otherwise stated, *A. thaliana* (L.) Heynh plants were grown on half-strength Murashige and Skoog agar media (Murashige & Skoog, 1962) without sucrose for 6 d under the specified entrainment conditions at 22°C. Total light intensity from both the red and blue LEDs (NIPHT, Edinburgh, UK) was 40–60 μmol m^−2^ s^−1^ (50% each colour). Luciferin was applied, as previously described (McWatters *et al.*, 2007), 24 h before imaging started. Plants were separated by opaque barriers and/or by sufficient distance on the imaging plates to minimize crosstalk by light scattering.

### *A. thaliana* imaging conditions

Light emitted from luciferase was measured using slow-scan, cooled charge-coupled device cameras (Orca II BT 1024; Hamamatsu Photonics, Hamamatsu, Japan), with 30 min exposures being collected (in the dark) following a delay of 1.25 h. Full technical details, including the design of an imaging dark suite, are available on the laboratory website: http://www.amillar.org/imaging.html. Lighting as required by the photoperiodic regime was formed of red and blue LEDs at a combined fluence rate of 40–50 μmol m^−2^ s^−1^ (50% each colour), controlled through Wasabi software (Hamamatsu Photonics). Images were collected following a 5 min delay to avoid autofluorescence. The same software script controlled the imaging and the change in light conditions. For both species, a long day (LD) was 16 : 8 h, light : dark, and a short day (SD) was 8 : 16 h, light : dark.

### *Ostreococcus tauri* growth and assay conditions

*Ostreococcus tauri* (Courties et Chretiennot-Dinet) OTTH0595 cells were cultured as described (O'Neill *et al.*, 2011) under the photoperiods indicated. The artificial seawater was refreshed and luciferin was applied 24 h before assays started. Cultures were transferred at dawn on the first day of bioluminescent assays to TopCount counters (Perkin-Elmer, Seer Green, UK). The photoperiod was controlled through an automated timing switch that regulated red and blue LEDs (NIPHT) with a combined fluence rate of 10 μmol m^−2^ s^−1^ (65% blue, 35% red). Luminescence was measured after 2 min darkness to avoid autofluorescence.

### Data analysis

Bioluminescence intensities from *A. thaliana* imaging were quantified using Metamorph software (MDS, Toronto, ON, Canada). Rhythmicity for both *A. thaliana* and *O. tauri* luminescence reads was analysed using the software package BRASSv3 (Edwards *et al.*, 2010), available from www.amillar.org. All experimental data were normalized to the average luminescence count for each time series. Phase and interpeak differences were measured through the manual quantification of peak times from raw data. This allowed subtle, secondary peak differences to be measured, which would otherwise have been masked in computational analysis.

### Model and data availability

The *O. tauri* models are available from the PlaSMo resource (www.plasmo.ed.ac.uk) and the Biomodels database, and the data are available from the BioDare data repository (www.biodare.ed.ac.uk; Moore *et al.*, 2014) with the accession numbers described in Table S1.

### Computational modelling

#### Analysis of light inputs to the *O. tauri* model

Parameters controlling light inputs to the model (Troein *et al.*, 2011) were fixed in turn, and the remaining model parameters were reoptimized to match training data (Troein *et al.*, 2011). Model performance was assessed using the scaled error (Troein *et al.*, 2011), where the error for each experiment was normalized relative to the error of a flat line through the data mean. Any useful model would have a total cost well below 1. The reference parameter set had a cost of 0.391 (Troein *et al.*, 2011); the change in error of the reoptimized models is expressed as a percentage of this.

#### Measuring clock model flexibility

The method that was used to quantify a circadian network's flexibility is based on analysing the linearization of the map relating parameter variations to changes in circadian outputs, as detailed in Rand *et al*. (2004, 2006) and Rand (2008). Here, we give an overview of the underlying theory.

For a given model, the method analytically determines the effect of varying the kinetic parameters *k *= (*k*_1_, …, *k_s_*) on the periodic solution *γ* = (*γ*_1_, …, *γ_d_*), representing the free-running or entrained clock. The components *γ*_*i*_ = *γ*_*i*_(*t*) are the expression time series of the network's constituent mRNAs and proteins. *γ* thus defines the clock model's free-running or entrained limit cycle (Rand *et al.*, 2004, 2006; Rand, 2008; Akman *et al.*, 2010).

Each variation of the parameters, δ*k *= (δ*k*_1_, …, δ*k_d_*), results in a corresponding variation to the limit cycle, δ*γ* = (δ*γ*_1_, …, δ*γ_d_*). For the free-running system, δ*γ* will primarily be manifested as a change in oscillation period, and in the phases of the clock's components relative to one another. For the entrained system, δ*γ* will primarily be manifested as a change in the components' phases relative to dawn and dusk (Rand *et al.*, 2004, 2006; Rand, 2008; Akman *et al.*, 2010). If the parameter changes are small, this variation is approximately given byEqn 1
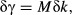


where the elements *M*_*ij*_ of the matrix *M* are the partial derivatives ∂*γ*_*i*_/∂*k*_*j*_. An ensemble of perturbations {δ*k*^(*l*)^} is considered, where the 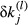
values are taken to be zero-mean independent identically distributed random variables. Geometrically, this ensemble can be thought of as a ball containing the set of all possible parameter changes resulting from stochastic evolutionary processes of a bounded size. This ball is mapped by *M* to an ellipsoid containing all the possible corresponding changes to the limit cycle, {δ*γ*^(*l*)^}. The singular values *σ*_1_ ≥ *σ*_2_ ≥ … ≥ *σ*_*s*_ ≥ 0 of *M* specify the lengths of this ellipsoid's principal axes, and so quantify the extent to which the limit cycle can be tuned by parameter variations. Large singular values correspond to the most flexible (or evolutionarily accessible) limit cycle components, for which significant changes can be obtained with small changes to the parameters. Conversely, small singular values indicate stiff components which require comparatively larger parameter changes to modify their behaviour.

It follows that the flexibility of a clock model can be characterized by its flexibility dimension, *d*, defined formally as the number of singular values lying above a chosen threshold, *ε*. This quantity specifies the number of key system outputs that can be simultaneously tuned by evolutionary processes, thereby providing a tractable method for comparing the flexibility of different clock models (Rand *et al.*, 2004, 2006; Rand, 2008).

In order to account for the fact that the values of clock model parameters and components can span several orders of magnitude, it is appropriate to consider relative parameter perturbations, δ*k*_*j*_/*k*_*j*_, and relative limit cycle perturbations, δ*γ*_*i*_/*γ*_*i*_ (Rand *et al.*, 2004). For each model, the singular value spectrum was therefore computed from the matrix M* = ((*k*_*j*_*/γ*_*i*_)*M*_*ij*_) using the numerical method described in Akman *et al*. (2010). Flexibility dimensions were calculated with *ε* set at 0.01 after scaling the singular value spectra by the largest leading singular value *σ*_1_ of the entrained models (Rand *et al.*, 2006; Rand, 2008).

Finally, in relating the calculated flexibilities to the number of feedback loops and light inputs, a feedback loop was defined to be a simple directed cycle in a model's network graph, while a model component was considered to have a light input if its expression was directly modulated by the light : dark cycle (Troein *et al.*, 2009).

#### Dusk sensitivities and phase flexibility

Dusk sensitivities are a concise index for measuring the phase changes of simulated mRNA and protein expression patterns in response to photoperiod variations (Akman *et al.*, 2010; Edwards *et al.*, 2010). For a given clock model component *γ*_*i*_, changing the time of dusk *T*_D_ by Δ*T*_D_ and the time of dawn *T*_L_ by Δ*T*_L_ results in a change Δ*φ*_*i*_ in the phase *φ*_*i*_, which is approximated by:Eqn 2
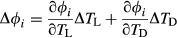


Here, the partial derivative ∂*φ*_*i*_/∂*T*_D_ defines the component's dusk sensitivity and ∂*φ*_*i*_/∂*T*_L_ its dawn sensitivity. These sensitivities sum to 1, meaning that their magnitudes indicate the relative responses of *φ*_*i*_ to changes to dusk and dawn (Akman *et al.*, 2010; Edwards *et al.*, 2010). In particular, a dusk sensitivity of 1 implies a dawn sensitivity of 0; this corresponds to the component's phase being exactly locked to dusk, since, from Eqn 2, Δ*φ*_*i*_ will be equal to Δ*T*_D_ (and independent of Δ*T*_L_). Conversely, a dusk sensitivity of 0 implies a dawn sensitivity of 1, implying exact locking to dawn. A dusk sensitivity that lies between 0 and 1 indicates a more flexible phase response, with *φ*_*i*_ exhibiting responses to both dusk and dawn changes. Here, a given phase *φ*_*i*_ is considered dusk-tracking if ∂*φ*_*i*_/∂*T*_D_ > 0.75, dawn-tracking if ∂*φ*_*i*_/∂*T*_D_ < 0.25 and flexible if 0.25 < ∂*φ*_*i*_/∂*T*_D_ < 0.75.

For all models, dusk sensitivities were computed as previously described (Akman *et al.*, 2010).

#### Model simulations and software

All model simulations and numerical analyses were carried out with custom software developed in MATLAB (Mathworks, Cambridge, UK), which is available on request. The SaSSy software package also calculates flexibility dimension and is available from http://www2.warwick.ac.uk/fac/sci/systemsbiology/.

## Results

### *O. tauri* shows rapid phase adjustment in bulk protein abundance to changes in photoperiods

**Fig 1:**
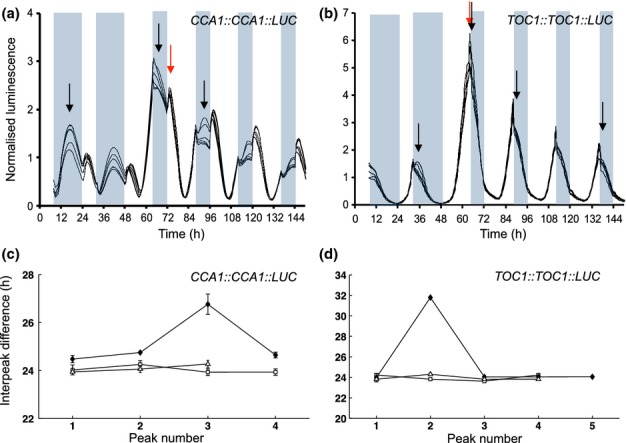
*Ostreococcus tauri* core circadian markers respond quickly to photoperiod transitions. *O. tauri* cells expressing constructs of CIRCADIAN CLOCK ASSOCIATED 1 (CCA1) or TIMING OF CAB 1 (TOC1) tagged with the marker gene LUCIFERASE (LUC) were entrained under 8 : 16 h, light : dark cycles (short days, SD) and imaged for 2 d: (a) *CCA1*::*CCA1*::*LUC*; (b) *TOC1*::*TOC1*::*LUC*. At 48 h, photoperiod conditions were switched to 16 : 8 h, light : dark cycles (long days, LD). All traces are normalized to the average of each time series and then averaged across eight replicate cultures. Red arrows highlight acute responses to light; black arrows denote circadian peaks. Interpeak differences between circadian peaks were calculated for SD (open squares), LD (open triangles) and SD to LD (closed diamonds) photoperiod conditions, for *CCA1::CCA1::LUC* (c) and *TOC1::TOC1::LUC* (d). White (day) and grey (night) shading represent the photoperiods in (a) and (b), respectively.

To investigate the re-entrainment of core clock components, *O. tauri* cell cultures carrying transgenic transcriptional/translational reporters for *O. tauri* circadian clock genes were moved from constant SD (8 : 16 h, light : dark) entrainment to constant LD (16 : 8 h, light : dark) entrainment. The clock reporters were monitored for 4.5 further cycles. In each case, the timing of reporter gene expression closely matched that of controls held in constant LD (Fig. S2) by the end of the experiment, indicating stable re-entrainment. The CCA1 circadian peak occurred in the night under SD conditions (black arrow, Fig. 1a). The peak was delayed by *c*. 2 h after the SD to LD transition, keeping the peak within the night phase and increasing the interpeak time difference on this cycle (Fig. 1c). The peak time in LD was similar to the first peak in continuous light (LL) at 20.7 ± 0.1 h (Fig. S3a). The acute light response of CCA1 produced a second peak *c*. 2 h after dawn (Fig. 1a, red arrow) and a small decrease in CCA1 protein abundance at dusk, consistent with earlier studies (Corellou *et al.*, 2009; Djouani-Tahri *et al.*, 2010; van Ooijen *et al.*, 2011; Troein *et al.*, 2011).

*Ostreococcus tauri* TOC1 protein was also strongly regulated by light : dark transitions (Fig.1b). Its higher degradation rate in darkness (Djouani-Tahri *et al.*, 2010; van Ooijen *et al.*, 2011) resulted in a sharp fall in TOC1 after the light to dark transition (red arrows in Figs1b, S2c,g). TOC1 continued to accumulate throughout the first LD, reaching nearly threefold higher values compared with SD and delaying the peak in bulk TOC1 protein abundance until the later light : dark transition (64 h, Fig. 1b). This is reflected in a large interpeak difference of *c*. 8 h (Fig. 1d). The circadian peak in TOC1 occurred at 16.8 ± 0.2 h under constant light (Fig. S3c), much later than in SD and 4 h before the peak in CCA1 (Fig. S3a). This peak time would fall just after dusk in LD. Consistent with this, the circadian peak of TOC1 in LD appeared 2 h or less after dusk, as a shoulder on the falling phase that is more distinct in some traces (highlighted by black arrows in Fig. 1b). A very similar shoulder followed dusk in SD (10 and 34 h; Fig. 1b).

The regulation of TOC1 and CCA1 were distinct, demonstrating that the stability of the reporter protein, luciferase, was not the major regulatory factor being observed. The *CCA1* reporters and *TOC1* transcriptional reporter lines showed acute light induction after dawn under light : dark cycles (Figs1, S2, S4, denoted by the red arrows), but not constant light (Fig. S3). These features are therefore a response to changes between light and dark conditions, which may contribute to entrainment (see the Discussion section).

**Fig 2:**
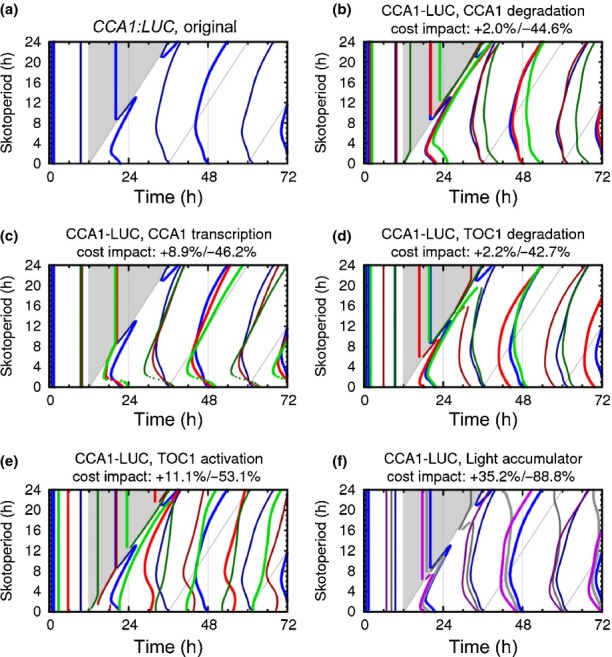
Modelling the effects of individual light inputs on *Ostreococcus tauri* phase in perturbed light conditions. The system was entrained in 12 : 12 h, light : dark cycles and released into continuous light (LL) after a single night of varying length (Skotoperiod; shaded area). Peaks (bright lines) and troughs (dark lines) are shown as functions of time and the timing of dawn. (a) The original model (blue), also included in all panels. (b–e) The effects of removing light inputs by fixing one light-dependent parameter to the value it would normally hold in the light (red) or dark (green): (b) CIRCADIAN CLOCK ASSOCIATED 1 (CCA1) protein degradation rate (*D*_c,l_ and *D*_c,d_ in the equations); (c) activation of *CCA1* transcription by (TIMING OF CAB 1) TOC1 (*R*_c,l_/*R*_c,d_); (d) TOC1 protein degradation rate (*D*_t,l_/*D*_t,d_); (e) activation rate of TOC1 protein (*K*_t,l_/*K*_t,d_). (f) Removing the ‘light accumulator’ effect on *TOC1* transcription, so *TOC1* is expressed as if in constant light (grey) or modulated directly by light without an accumulator (purple). The numbers above the panels show the impact of each light input on the overall model fit, expressed as the change in the cost function after model alteration and local parameter refitting, relative to the lowest cost obtained with all or no light inputs. The first number is the increase in cost if the light input is removed from the full model, and the second is the decrease in cost if the light input is added to the model with no light inputs.

The entraining effects of individual light-sensitive processes cannot yet be dissected experimentally, so they were tested in the detailed *O. tauri* clock model (Troein *et al.*, 2011). First, an experimental protocol that compared the effect of many different light regimes was simulated, in models that lacked one of the five light inputs. In the experiments, *CCA1:CCA1:LUC O. tauri* cultures entrained in 12 : 12 h, light : dark cycles were exposed to a final night of 2–22 h duration and transferred to constant light (Troein *et al.*, 2011). The phase of the rhythm in constant light was not locked to either dawn or dusk, and the model simulations closely matched the data (Fig.2a; Troein *et al.*, 2011), providing one of the clearest indications that the *O. tauri* clock supported flexible entrainment. When the dawn induction of *CCA1* transcription was removed by setting the relevant parameter constitutively to its value in the light or in the dark, the simulated phase in constant light tracked dawn more strongly (Fig.2c), clearly departing from the original model and the experimental result (Fig.2a). The other light inputs gave different effects (Fig.2), so light activation of *CCA1* transcription made a distinctive contribution. Secondly, the contribution of each light input to the clock circuit's dynamic behaviour was tested. The parameters of each model lacking one light input were reoptimized to fit all 144 time series used in model construction (Troein *et al.*, 2011). The best fit of the model lacking the light induction of *CCA1* transcription was 8.9% worse than the full model (values shown in Fig.2). This light input contributes to matching the data, but less than the activation of *TOC1* transcription by the light accumulator, which integrates light over the past 24 h to control average expression levels (35% worse fit). Thirdly, the potential contribution of each light input alone was tested by reoptimizing the parameters of a model from which all other light inputs were removed (values shown in Fig.2). Each of the four acute light inputs improved the fit to the training data by 42–53%, which suggests that any mechanism of light input is better than none.

### *A. thaliana* shows dual, light and circadian responses to changing photoperiod

**Fig 3:**
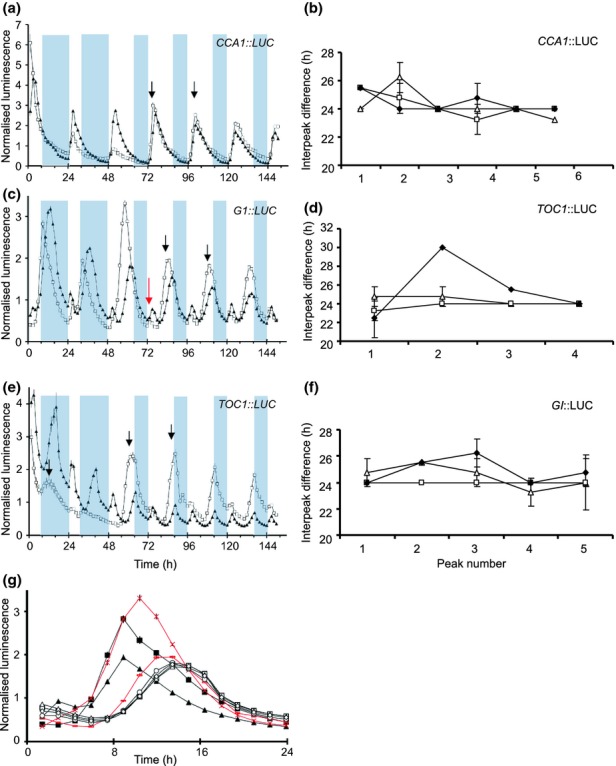
*Arabidopsis thaliana*'s response to the short day (SD) to long day (LD) transition. *A. thaliana* plants containing luciferase (LUC)-tagged clock gene constructs were entrained under white light for 6 d in SD (8 : 16 h blue and red light : dark cycles), and following 2 d of imaging conditions were switched to LD (16 : 8 h blue and red light : dark cycles) and imaged for a further 4 d (open squares). Controls were entrained and imaged under LD (closed triangles). Error bars on luminescence plots denote ± SE of the mean (*n *=* *12–16). Interpeak differences were calculated from circadian peaks (black arrows); errors represent average interpeak difference from two biologically independent experiments; SD, open squares; LD, open triangles; SD to LD, closed diamonds. (a) CIRCADIAN CLOCK ASSOCIATED 1, *CCA1*::*LUC*; (b) *CCA1*::*LUC* interpeak difference; (c) TIMING OF CAB1, *TOC1*::*LUC*; (d) *TOC1*::*LUC* interpeak difference; (e) GIGANTEA,* GI*::*LUC*; (f) *GI*::*LUC* interpeak difference. (g) Individual day traces for *GI*::*LUC*: day 1, black squares; day 2, black triangles; day 3, red crosses; day 4, red dashes; day 5, white circles; day 6, white diamonds; day 7, white squares; day 8, white triangles. ZT, Zeitgeber Time (hours since lights-on).

To investigate the re-entrainment of a circadian network possessing multiple feedback loops, the same SD to LD photoperiod transition was applied to *A. thaliana*. The peak phase of *CCA1* after dawn showed a *c*. 1 h delay in LD compared with SD conditions, but this was within the variation observed for *CCA1* peak times under stable photoperiods (Fig.3b). This result indicates that the *CCA1* phase was largely locked to dawn: an 8 h change in day-length at dusk had little impact on its peak time (Fig.3a,b). Plants grown under constant LD showed a peak in *CCA1 c*. 2 h later than plants grown under constant SD (Edwards *et al.*, 2010). A gradual shift of *c*. 2 h is observed in the *GI* circadian peak (Fig.3e; quantified as interpeak difference in 3f; individual seedling traces in Fig. S5). *GI* expression also showed an acute light response at dawn (highlighted by a red arrow in Fig.3e), consistent with previous results (Locke *et al.*, 2005b; Edwards *et al.*, 2010). The progressive shift in the circadian phase of *GI* can be clearly observed when the traces of successive days are superimposed (Fig.3g; red crosses mark the transition day, day 3). Continued illumination on the transition day led to a 20% higher peak of *GI* expression, which was delayed by 1.5 h. The first subsequent day (day 4) brought a further phase delay and a reduction in amplitude, owing to the concomitant changes in other regulators of *GI*. Expression on day 5 was still slightly advanced compared with the final phase on days 6 and 7 of this experiment (Fig.3g).

*Arabidopsis thaliana TOC1* showed a very similar pattern of expression to that of *O. tauri*, with strong light responses that yielded a sharp, single shift of *c*. 8 h in interpeak differences (Fig.3c,d). The *A. thaliana TOC1* RNA had rhythmic regulation of lower amplitude than the *CCA1* and *GI* transcripts (Edwards *et al.*, 2010). The lower amplitude of the *TOC1* reporter rhythm observed under SD compared with LD conditions is also consistent with previous results (Perales & Más, 2007; Edwards *et al.*, 2010). Under light : dark cycles we found significant acute light responses, making the circadian regulation harder to identify (examples of circadian peaks under SD to LD transitions are highlighted in Fig.3c).

### Flexibility within the modelled circadian networks is dependent on both the number of loops and the number of light inputs

Some clock components in *A. thaliana* and *O. tauri* showed similar re-entrainment after the photoperiod transition, suggesting that each network's dynamics was not determined simply by its number of feedback loops. To test which features of the networks might provide the required regulation, each model's flexibility was quantified using its flexibility dimension, *d*, a global measure of the extent to which the waveforms of all simulated mRNA and protein expression time series can be tuned by varying the model's kinetic parameters (Rand *et al.*, 2006; Rand, 2008). These parameters quantify the biochemical processes underlying the network dynamics, such as transcription, translation, phosphorylation, degradation and the cooperativity of substrate binding.

**Fig 4:**
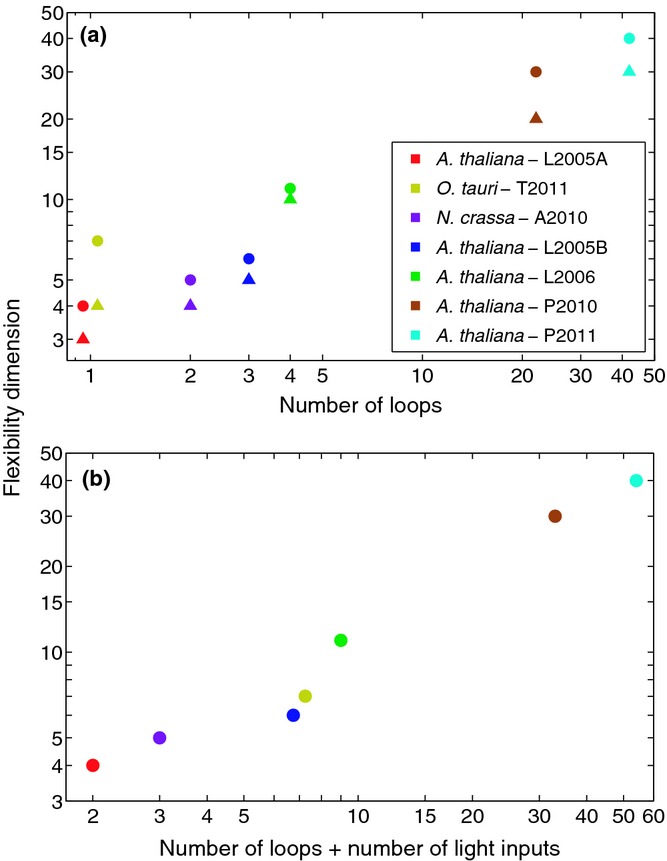
Variations in clock flexibility with feedback loops and light inputs. Circles denote the flexibility dimension *d* of a model in 12 : 12 h, light : dark cycles, while triangles denote the flexibility under constant light conditions (continuous light for *Arabidopsis* and *Ostreococcus*; continuous dark for *Neurospora*). (a) Flexibility increases with loop number under constant conditions. In each case, the computed value of *d* is higher in light : dark cycles than in constant light conditions, showing that incorporating light entrainment yields a more flexible circuit. In particular, the single-loop *O. tauri* circuit (T2011) exhibits a significant increase in flexibility compared with the single-loop *A. thaliana* circuit (L2005A). (b) Plotting flexibility against the sum total of loops and light inputs for each model reveals a positive trend, implying that light inputs augment clock flexibility in a similar manner to feedback loops. (Note that in both plots, points with the same *x*-axis value have been slightly offset for clarity.) For all models, *d* is defined as the number of significant singular values of the matrix M* that maps parameter perturbations to perturbations of the corresponding free-running or entrained limit cycle (the singular value spectra {*σ*_*k*_} of the entrained models are shown in Fig. S7).

The comparison of the calculated flexibility dimensions of the *A. thaliana* and *O. tauri* models in constant conditions showed that flexibility increases with the total number of feedback loops in the circuit (Fig. 4). To increase the generality of this analysis, a two-loop model of the *N. crassa* clock (A2010; Fig. S1c) was also included (Akman *et al.*, 2010). When entrainment was incorporated, the flexibility of each model network also increased. Fig. 4 shows that in LL, the flexibility of the single loop *O. tauri* (T2011) network (Troein *et al.*, 2011) is close to that of the single loop *A. thaliana* (L2005A) network (Locke *et al.*, 2005a). However, the addition of light : dark cycles to *O. tauri* results in a flexibility that is intermediate between the multiloop *A. thaliana* L2005B (Locke *et al.*, 2005b) and L2006 (Locke *et al.*, 2006) circuits.

### With increasing flexibility, more core circadian components show dual light and circadian regulation

**Fig 5:**
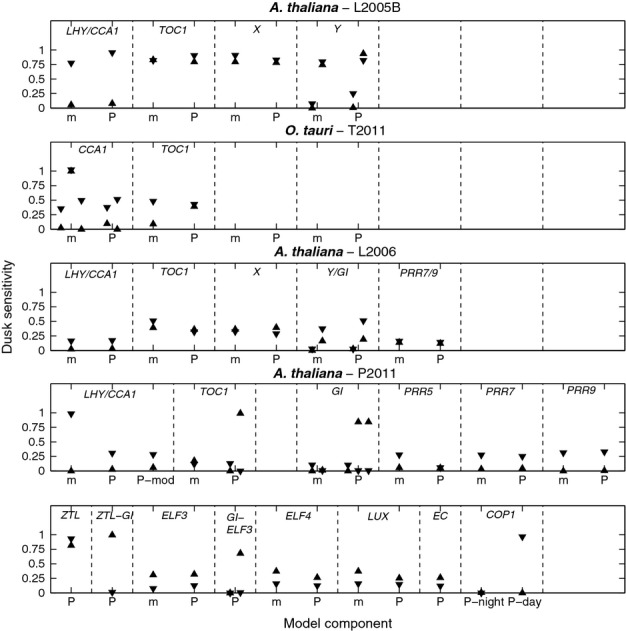
Dusk sensitivities measure phase flexibility. Dusk sensitivities for the peak (upward triangle) and trough (downward triangle) times of mRNA (m) and bulk protein (P) variables of all genes in four of the most flexible models. These were calculated from simulations of the model in 12 : 12 h, light : dark cycles in each case. A dusk sensitivity close to 1 indicates dusk-dominant entrainment; one close to 0 indicates dawn-dominant entrainment. Intermediate values denote flexible entrainment, in which both dawn and dusk information is integrated. Where an expression profile has multiple peaks or troughs, dusk sensitivities are plotted from left to right, in chronological order after dawn, with the convention that in each peak/trough pair, the trough follows the peak in time. While the *Arabidopsis*L2005B circuit exhibits only dawn or dusk locking, the more flexible *Ostreococcus*T2011 and *Arabidopsis* L2006 models have several components with intermediate light responses (e.g. the trough of *TOC1* mRNA which tracks the middle of the night in both models). The most flexible circuit, *Arabidopsis*P2011, exhibits the broadest spectrum of dusk sensitivities. The positive correlation between network and phase flexibility is quantified further in Fig. S6.

Dusk sensitivity (Akman *et al.*, 2010) is a measure of how the pattern of entrainment for any circadian rhythm responds to a change in photoperiod, and was applied to analyse the *A. thaliana* and *O. tauri* clock models (Fig. 5). Comparing the dusk sensitivity spectra of the models indicated that the number of components of a model possessing flexible, entrained phase responses is positively correlated with the model's flexibility dimension (Fig. S6). Indeed, the highly flexible P2011 and T2011 architectures each lock the peak/trough times of some components to dawn or dusk, whilst others exhibit greater flexibility. In *CCA1* regulation, for example, mRNA peaks in both circuits are exclusively locked to either dusk or dawn, while protein troughs respond more flexibly to changes in both these light signals.

## Discussion

For a circadian mechanism to be beneficial, the phase of its components must be entrained appropriately to the environmental day : night cycle (Dodd *et al.*, 2005). The entrained phase of circadian rhythms changes progressively with day length, in *O. tauri*,* A. thaliana* and other organisms. Under light : dark cycles, core circadian clock components show acute light regulation (Fig. S2) that is expected to contribute to entrainment (Edwards *et al.*, 2010). The experiments and analysis presented in this paper tested the specific responses of these components to an environmental transition of photoperiod between SD and LD, to identify the general features of clock systems that support flexible entrainment.

In *O. tauri*, a change in photoperiod, tested experimentally through the shift from SD to LD, immediately affected the measured circadian components, with rapid shifts observed in the peak phases of CCA1 and TOC1 proteins. The TOC1 protein waveform responded immediately to the altered time of the light to dark transition, consistent with other reports (Corellou *et al.*, 2009; Djouani-Tahri *et al.*, 2010; van Ooijen *et al.*, 2011; Troein *et al.*, 2011). The circadian peak overlapped with the light : dark-driven peak under LD conditions (Fig. 1b,d). The circadian peak in CCA1 protein abundance shifted past the middle of the (shorter) night in the second LD cycle (Fig. 1a,c). This pattern suggests strong light responses relative to the amplitude of the circadian limit cycle, such that light input rapidly and completely reset the clock (Johnson *et al.*, 2003).

The acute light response in *O. tauri CCA1* transcription at dawn (Fig. S2a, red arrow) was required for the detailed clock model to match the observed pattern of entrainment (Fig.2; Troein *et al.*, 2011), suggesting that it, or an equivalent feature, is functionally relevant. However, not all features of LUC reporter waveforms *in vivo* are easy to detect by biochemical assays, especially if small, acute light responses overlap a larger circadian peak (Corellou *et al.*, 2009; Edwards *et al.*, 2010), so a contribution of reporter-specific effects to the waveform remains possible.

In *A. thaliana*, strong light responses were also observed. The *TOC1* waveform quickly shifted with the photoperiod change, merging with the light : dark transition on the second cycle of LD. The acute light response in the *TOC1* reporter at dawn has been observed in some past studies (such as Dodd *et al.*, 2014) but not others (such as Edwards *et al.*, 2010). *A. thaliana GI* peaked at the end of the day in SD, when acute light activation ceased (Fowler *et al.*, 1999; Locke *et al.*, 2005b; Edwards *et al.*, 2010). *GI* expression peaked within the light interval under LD, avoiding overlap with light : dark transitions, and took three cycles to alter phase stably (Fig.3g). Such transient cycles are commonly observed in circadian systems (Johnson *et al.*, 2003), as the molecular components of the clock move from one limit cycle to another. The SD to LD transition in *A. thaliana* caused similar transient cycles to the transition from altered entrainment regimes to constant light (Dodd *et al.*, 2014). The longer duration of transients in *A. thaliana* than in *O. tauri* is consistent with the need for many more components in the *A. thaliana* clock network to find new, stable phase relationships, whereas the relatively large number of light inputs in *O. tauri* quickly resets its few clock components.

Model analysis of the *O. tauri* and *A. thaliana* networks revealed that in constant light conditions, flexibility increases with the number of feedback loops (Fig. 4). This is consistent with previous theoretical work suggesting that the flexibility of a general clock network will be roughly proportional to its loop complexity (Rand *et al.*, 2004, 2006; Rand, 2008). In entrained conditions, however, flexibility was correlated with the total number of feedback loops and light inputs. Indeed, for *O. tauri*, the high flexibility observed in light : dark cycles was not accounted for by the network's loop topology. Rather, the five light inputs to its single feedback loop generated a similar degree of flexibility to an *A. thaliana* circuit comprising three feedback loops and five light inputs (cf. Figs4, S1).

Analysis of the dusk sensitivities of the clock components in the models (Figs5, S6) further revealed that the increased network flexibility conferred by multiple feedback loops and/or light inputs is partly manifested as greater flexibility of entrained phase, confirming the predictions made in earlier studies (Akman *et al.*, 2010; Edwards *et al.*, 2010). This analysis was consistent with the dual light and clock regulation of individual clock components observed experimentally in both species; whilst certain components were locked to either dawn or dusk (e.g. peak *CCA1* expression in *A. thaliana*), others responded to both light signals, yielding a more graded response to photoperiod changes (e.g. the circadian peak of CCA1 protein expression in *O. tauri*). However, dawn/dusk locking in transcriptional and translational markers was much more prevalent in *O. tauri*, consistent with its faster entrainment, and contrasting with the greater flexibility of the more complex *A. thaliana* network. Some caution is warranted, as the five light inputs in the *O. tauri* model have not all been tested biochemically (Corellou *et al.*, 2009; van Ooijen *et al.*, 2011): they were the minimum set required to match a wide range of data (Troein *et al.*, 2011). However, each makes a distinctive contribution to the model's flexible entrainment (Fig.2). For comparison, 16 photoperiod-dependent parameters were adjusted to allow a simpler model of the *O. tauri* clock to match similar reporter data (Thommen *et al.*, 2012).

The importance of light inputs to the *O. tauri* clock might reflect an adaptation to gain regulatory flexibility, in the context of a very reduced genome size and a correspondingly simple loop structure. The complex clock circuits of larger eukaryotes are consistent with adaptation to noisy environmental conditions (Troein *et al.*, 2009; Domijan & Rand, 2011). By contrast, the simple *O. tauri* clock's dependence on light inputs might risk resetting to chance fluctuations. Rhythmically gated light inputs (Millar & Kay, 1996) offered a viable alternative in a single-loop model (Thommen *et al.*, 2010). Further model analysis will complement progressively more complex experimental tests and pioneering studies under field conditions (Nagano *et al.*, 2012), to understand how circadian timing controls environmental response pathways.

## Supporting Information

Fig S1 Circuit diagrams for the clock models. **Fig. S2** Core circadian markers in *O. tauri* under photoperiodic entrainment. **Fig. S3** *O. tauri* rhythms under constant light. **Fig. S4** Short day to long day transitions of *O. tauri* transcriptional markers. **Fig. S5** Reproducibility of short day to long day transition in *A. thaliana* for the *GIGANTEA* gene. **Fig. S6** Loop flexibility confers phase flexibility. **Fig. S7** Singular value spectra for the entrained clock models.

Table S1 Availability of models and data
