# Supplementary material for: Light and circadian regulation of clock components aids flexible responses to environmental signals
Source: New Phytol. 2014 May 20;203(2):568–77. doi: 10.1111/nph.12853 (PMC4286021; doi:10.1111/nph.12853)
Supplement: Fig S1 — Circuit diagrams for the clock models. Fig. S2 Core circadian markers in O. tauri under photoperiodic entrainment. Fig. S3 O. tauri rhythms under constant light. Fig. S4 Short day to long day transitions of O. tauri transcriptional markers. Fig. S5 Reproducibility of short day to long day transition in A. thaliana for the GIGANTEA gene. Fig. S6 Loop flexibility confers phase flexibility. Fig. S7 Singular value spectra for the entrained clock models. [file nph0203-0568-SD1.pdf]

Supporting Information Figures S1-S7

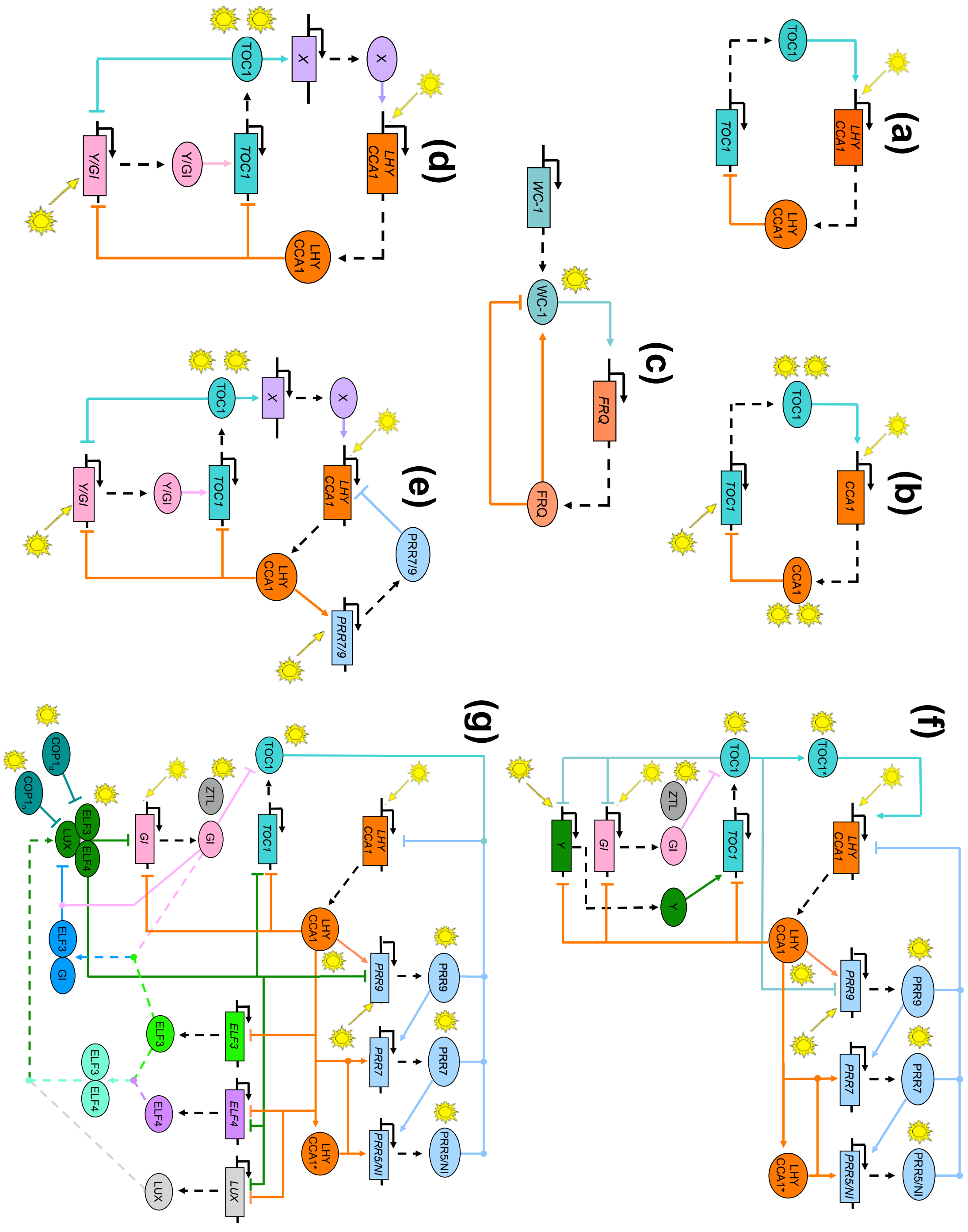

Fig. S1. Circuit diagrams for the clock models.

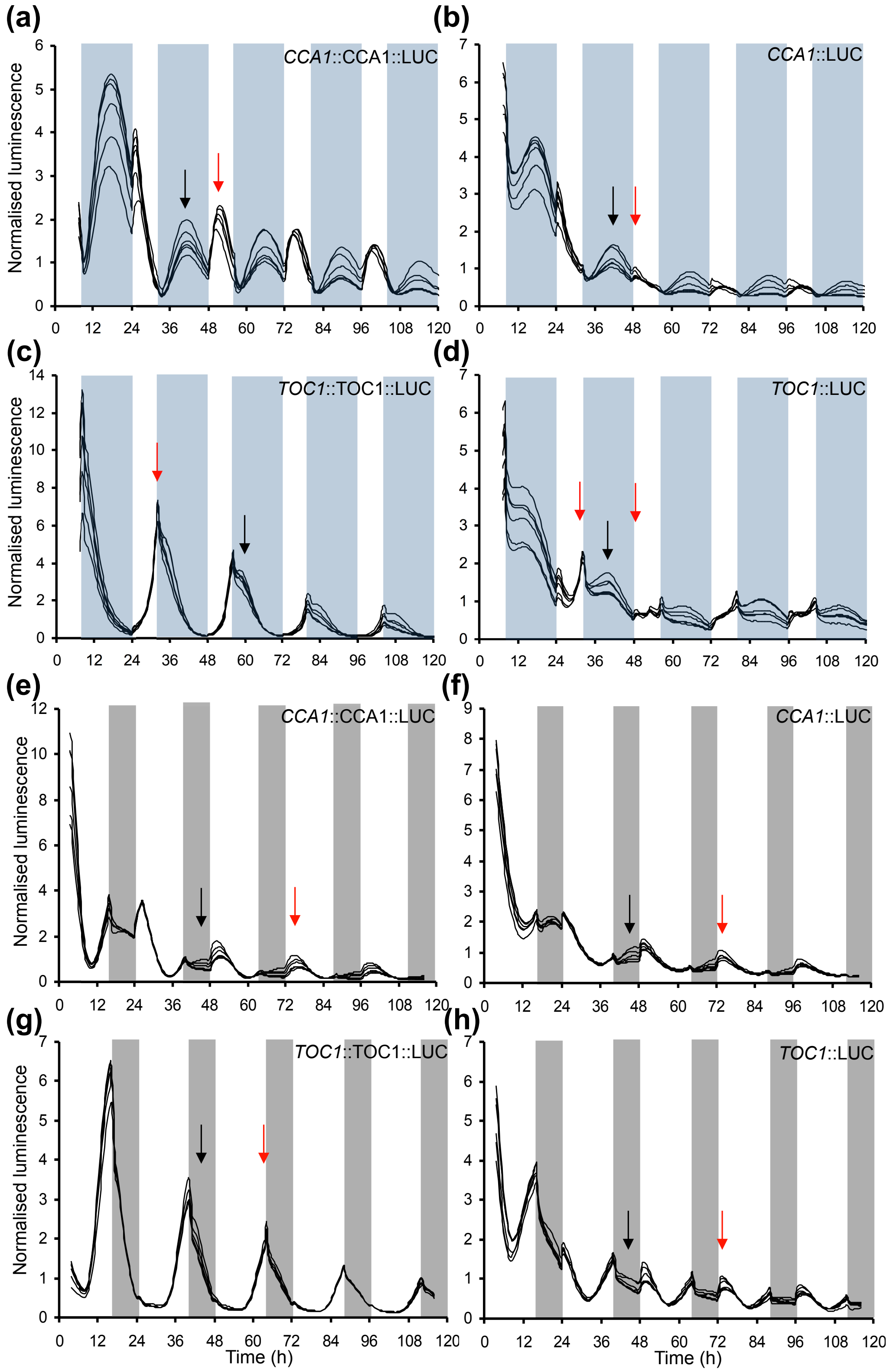

Fig. S2. Core circadian markers in *O. tauri* under photoperiodic entrainment.

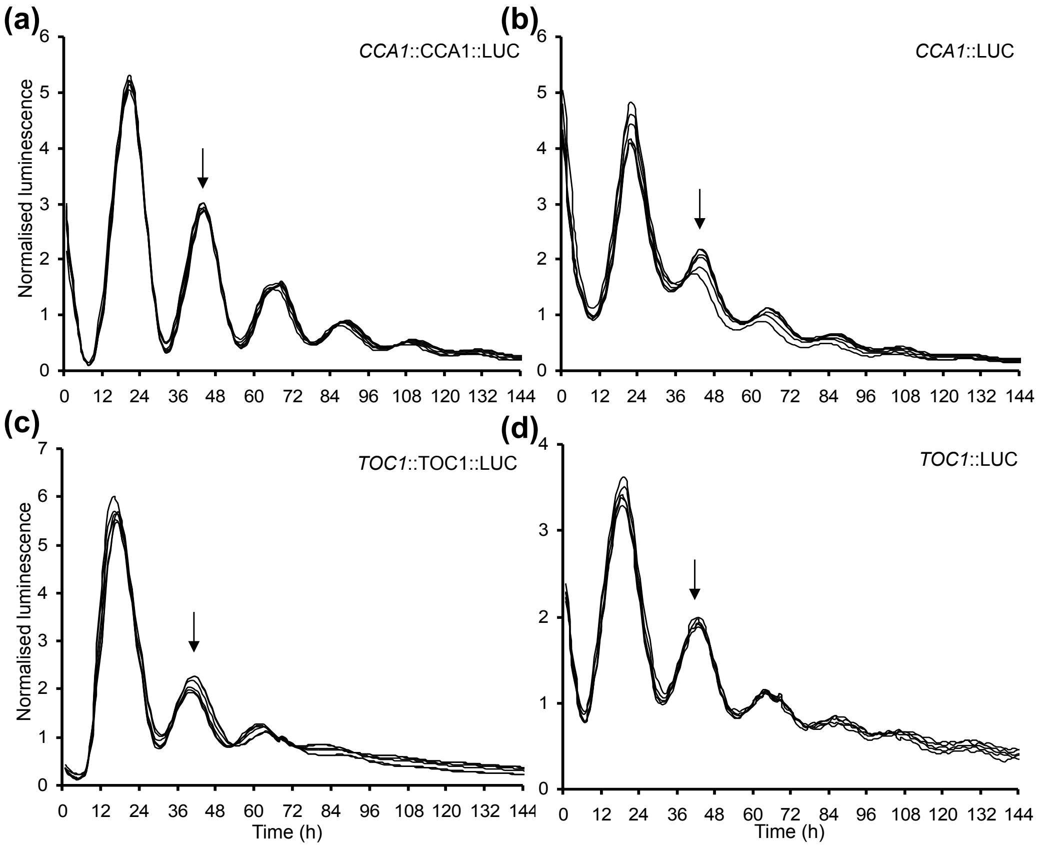

Fig. S3. *O. tauri* rhythms under constant light.

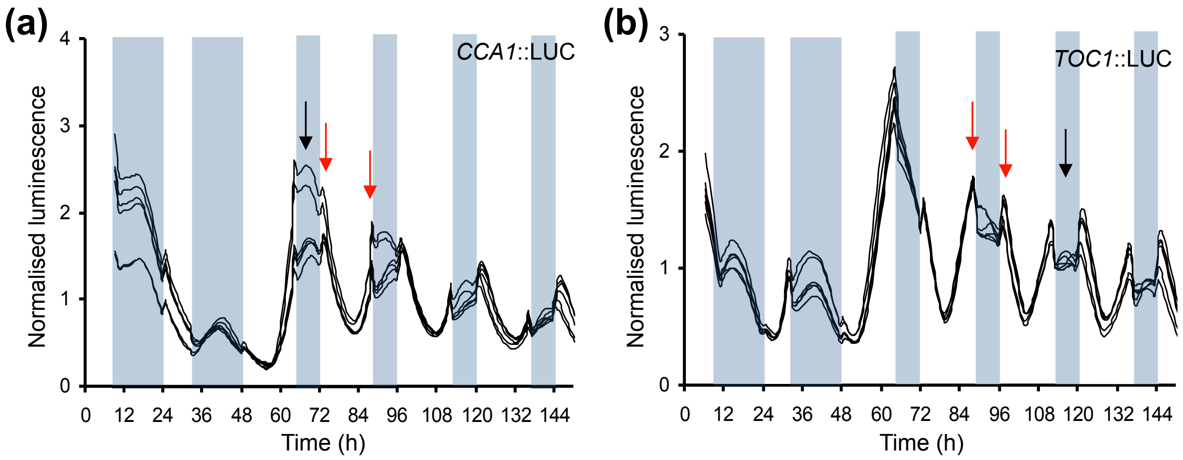

Fig. S4. Short day to long day transitions of *O. tauri* transcriptional markers.

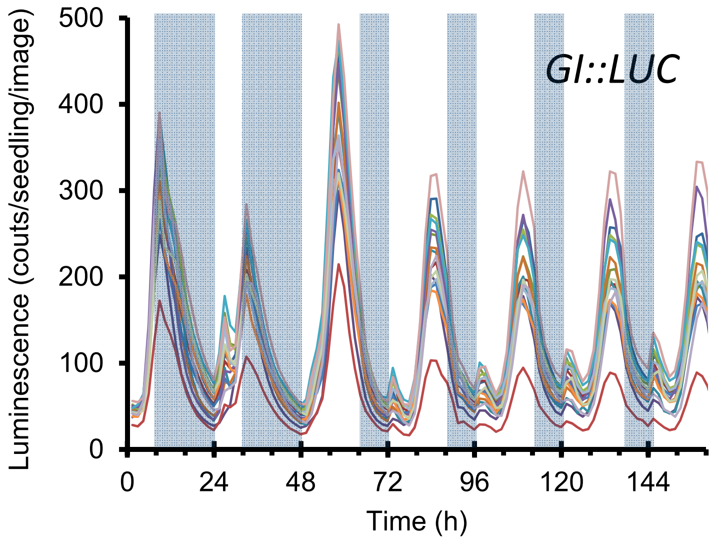

Fig. S5. Reproducibility of short day to long day transition in *A. thaliana* for thr GIGANTEA gene.

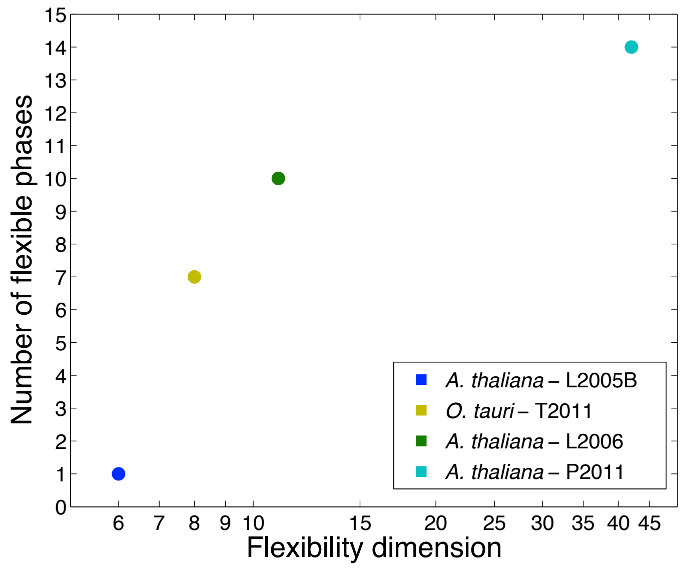

Fig. S6. Loop flexibility confers phase flexibility.

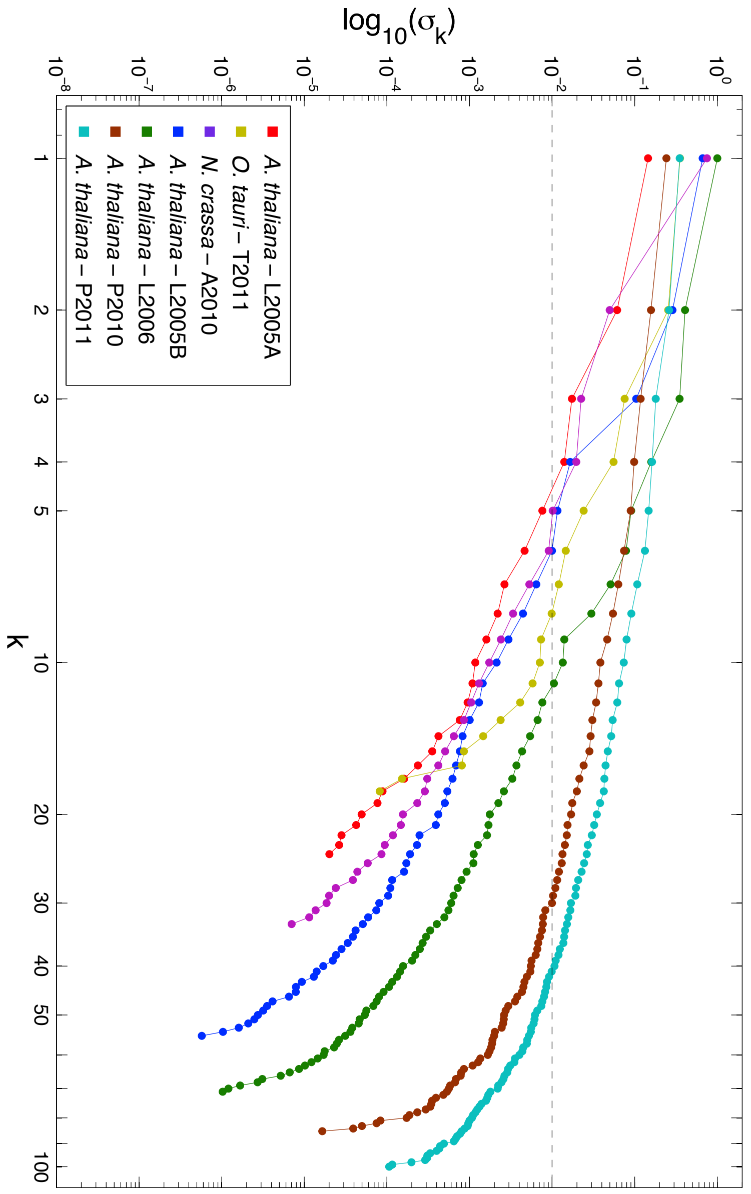

Fig. S7. Singular value spectra for the entrained clock models.

Figure Legend References

Akman OE, Rand DA, Brown PE, Millar AJ. 2010. Robustness from flexibility in the fungal circadian clock. *Biomedical Central Systems Biology* **4:** 88

Locke JC, Millar AJ, Turner MS. 2005a. Modelling genetic networks with noisy and varied experimental data: the circadian clock in *Arabidopsis thaliana*. *Journal of Theoretical Biology* **234:** 383-393

Locke JCW, Southern MM, Kozma-Bognár L, Hibberd V, Brown PE, Turner MS, Millar AJ. 2005b. Extension of a genetic network model by iterative experimentation and mathematical analysis. *Molecular Systems Biology* **1**:13

Locke JCW, Kozma-Bognar L, Gould PD, Feher B, Kevei E, Nagy F, Turner MS, Hall A, Millar AJ. 2006. Experimental validation of a predicted feedback loop in the multi-oscillator clock of Arabidopsis thaliana. *Molecular Systems Biology* **2:**59

Pokhilko A, Hodge SK, Stratford K, Knox K, Edwards KD, Thomson AW, Mizuno T, Millar AJ. 2010. Data assimilation constrains new connections and components in a complex, eukaryotic circadian clock model. *Molecular Systems Biology* **6:** 416

Pokhilko A, Fernández AP, Edwards KD, Southern MM, Halliday KJ, Millar AJ. 2012. The clock gene circuit in *Arabidopsis* includes a repressilator with additional feedback loops. *Molecular Systems Biology* **8:** 574

Rand DA, Shulgin BV, Salazar D, Millar AJ. 2004. Design principles underlying circadian clocks. *Journal of Royal Society Interface* **1:** 119-130

Rand DA, Shulgin BV, Salazar JD, Millar AJ. 2006. Uncovering the design principles of circadian clocks: mathematical analysis of flexibility and evolutionary goals. *Journal of Theoretical Biology* **238:** 616-635.

Rand DA. 2008. Mapping global sensitivity of cellular network dynamics: sensitivity heat maps and a global summation law. *Journal of Royal Society Interface* **5:** S59-S69.

Troein C, Corellou F, Dixon LE, Van Ooijen G, O'Neill JS, Bouget F-Y, Millar AJ. 2011. Multiple light inputs to a simple clock circuit allow complex biological rhythms. *Plant Journal* **66:** 375-385.
