# Supplementary material for: Light and circadian regulation of clock components aids flexible responses to environmental signals
Source: New Phytol. 2014 May 20;203(2):568–77. doi: 10.1111/nph.12853 (PMC4286021; doi:10.1111/nph.12853)
Supplement: Table S1 — Availability of models and data [file nph0203-0568-SD3.docx]

**Supporting Information Table S1 and legends to Figs S1–S7**

**Table S1 Availability of models and data**

Accession numbers for the *O. tauri* models on the PlaSMo resource, [www.plasmo.ed.ac.uk](http://www.plasmo.ed.ac.uk/), and for data on the BioDare data repository, [www.biodare.ed.ac.uk](http://www.biodare.ed.ac.uk/). BioDare experiments are retrieved by appending the experiment ID to the URL as follows (for experiment 12730740671132): <https://www.biodare.ed.ac.uk/robust/ShowExperiment.action?experimentId=12730740671132>. PlaSMo models are retrieved by appending the model ID to the URL as follows (for PLM_7): <http://www.plasmo.ed.ac.uk/plasmo/models/model.shtml?accession=PLM_7>. Either type of record can also be retrieved using the ID number with the resource’s Search function.

| **Asset name** | **Figure panels** | **ID** |
| --- | --- | --- |
| *O. tauri LUC* reporter data | 1a, 1b | 13982031569136 |
| *A. thaliana LUC* reporter data | 3a, 3d, 3e | 13982027442235 |
| *O. tauri* model, wild type T2011 | 2a | PLM_7 |
| *O. tauri* model, CCA1 degr L | 2b | PLM_85 |
| *O. tauri* model, CCA1 degr D | 2b | PLM_86 |
| *O. tauri* model, CCA1 prod L | 2c | PLM_87 |
| *O. tauri* model, CCA1 prod D | 2c | PLM_88 |
| *O. tauri* model, TOC1 degr L | 2c | PLM_89 |
| *O. tauri* model, TOC1 degr D | 2c | PLM_90 |
| *O. tauri* model, TOC1 act L | 2e | PLM_91 |
| *O. tauri* model, TOC1 act D | 2e | PLM_92 |
| *O. tauri* model, acc immediate | 2f | PLM_83 |
| *O. tauri* model, acc on | 2f | PLM_84 |

**Legends to Figs S1–S7**

**Fig. S1 Circuit diagrams for the clock models.**

Genes are boxed and arrows denote regulatory interactions. Light inputs modulating gene/protein expression are marked by sun symbols; double symbols indicate that light affects both the cytoplasmic and nuclear (or active and inactive) form of a protein. A) The *Arabidopsis* L2005A circuit (Locke *et al.,* 2005a). LATE ELONGATED HYPOCOTYL/CIRCADIAN CLOCK ASSOCIATED 1 (LHY/CCA1 protein) (combining LHY and CCA1 functions) represses production of the *LHY/CCA1* activator TIMING OF CAB1 (TOC1). Light activates transcription of *LHY/CCA1*. B) The *Ostreococcus* T2011 circuit (Troein *et al.,* 2011). This has the same basic architecture as A, with CCA1 repressing its activator TOC1. However in addition to upregulating *CCA1* transcription, light also (i) directly activates transcription of *TOC1*, (ii) augments the degradation of both CCA1 and TOC1 protein, and (iii) accelerates the rate at which TOC1 protein is converted into its active form. C) The *Neurospora* A2010 model (Akman *et al.,* 2010). FREQUENCY (FRQ) protein represses the *FRQ* activator WHITE COLLAR-1 (WC-1). FRQ also upregulates the production of WC-1, giving a positive feedback loop. Light activates *FRQ* transcription through a conformational change in WC-1. D) The *Arabidopsis* L2005B model (Locke *et al.,* 2005b). Compared to A, TOC1 activates *LHY/CCA1* indirectly through gene *X*, and *TOC1* is activated by light indirectly via gene *Y*. Y activates *TOC1* transcription and TOC1 represses *Y* transcription, yielding a separate evening loop. The degradation of cytoplasmic and nuclear TOC1 protein is dark-activated, mimicking the repressive action of ZEITLUPE (ZTL). E) The *Arabidopsis* L2006 model (Locke *et al.,* 2006). Compared to D, the additional PSEUDO RESPONSE REGULATOR 7/9 (*PRR7/9*) gene is light-activated, and its protein product represses *LHY/CCA1* transcription. LHY/CCA1 upregulates *PRR7/9*, giving a separate morning loop. F) The *Arabidopsis* P2010 circuit (Pokhilko *et al.,* 2010). Compared to E, this model separates the evening loop elements GIGANTEA (*GI*) and *Y*, whilst also incorporating the inhibition of TOC1 by GI via the (light-activated) stabilisation of ZTL protein. In addition, the morning loop is augmented to explicitly include both PRR9 and a night-inhibitor (NI) of *LHY/CCA1* expression for which PSEUDO RESPONSE REGULATOR 5 (PRR5) is a candidate component. The NI is upregulated by a modified form of LHY/CCA1 protein and undergoes dark-activated degradation, as do PRR7 and PRR9. Finally, LHY/CCA1 translation is assumed to be light-activated, and gene *X* is replaced by a post-translationally modified complex dependent on TOC1 protein, TOC1*, that is light-degraded. G) The *Arabidopsis* P2011 circuit (Pokhilko *et al.,* 2012). Compared to F, this model recasts the evening loop by removing gene *Y* and incorporating the evening complex (EC), formed from the binding of EARLY FLOWERING 3 (ELF3), EARLY FLOWERING 4 (ELF4) and LUX ARRHTYMIA (LUX proteins). The EC represses the expression of each of its constituent genes, as well as down-regulating *TOC1* and *PRR9* production, whilst itself being repressed by GI protein. The model also includes post-translational regulation of the EC by the ubiquitin E3 ligase CONSITUTIVELY PHOTOMORPHOGENIC 1 (COP1), which is assumed to exist in two inter-convertible forms, one of which has a higher concentration during the day, and the other a higher concentration at night.

**Fig. S2 Core circadian markers in *O. tauri* under photoperiodic entrainment.**

A-D) Cells expressing constructs of CIRCADIAN CLOCK ASSOCIATED 1 (CCA1) or TIMING OF CAB 1 (TOC1) tagged with the marker gene LUCIFERASE (LUC) were entrained and assayed under short days (SD). A) *CCA1::CCA1::LUC*, B) *CCA1*::*LUC*, C) *TOC1::TOC1::LUC*, D) *TOC1*::*LUC*. E-H) Cells were entrained and assayed under long days (LD). E) *CCA1::CCA1::LUC*, F) *CCA1*::*LUC*, G) *TOC1::TOC1::LUC*, H) *TOC1*::*LUC*. Traces are normalised to the average of each time series and then among replicates, *n*=6 for each condition. Red arrows indicate transient responses to light and black arrows indicate circadian responses. White (day) and grey (night) shading represents the photoperiod.

**Fig. S3 *O. tauri* rhythms under constant light.**

Cells expressing constructs of CIRCADIAN CLOCK ASSOCIATED 1 (CCA1) or TIMING OF CAB 1 (TOC1) tagged with the marker gene LUCIFERASE (LUC) were entrained under 12 h blue light :12 h dark cycles and released at ZT0 to constant red and blue light. A) *CCA1::CCA1::LUC*, B) *CCA1*::*LUC*, C) *TOC1::TOC1::LUC*, D) *TOC1*::*LUC*. Traces are normalised to the average of each time series with *n*=6 traces shown, representative of *n*=48. Black arrows indicate the circadian peak in each case.

**Fig. S4 Short day to long day transitions of *O. tauri* transcriptional markers.**

Cells expressing constructs of *CIRCADIAN CLOCK ASSOCIATED 1* (*CCA1*) or *TIMING OF CAB 1* (*TOC1*) promoters fused to with the marker gene *LUCIFERASE* (*LUC*) were entrained under short days (SD) and assayed from time 0h for 2 d. At time 48h, the photoperiod was switched to long days (LD). A) *CCA1*::*LUC* and B) *TOC1*::*LUC*. All traces are normalised to the average of each time series and then averaged across *n*=6 cultures, so the effects of light intensity do not affect the averages. Red arrows indicate transient responses to light, and black arrows indicate circadian responses. White (day) and grey (night) shading represents the photoperiod.

**Fig. S5 Reproducibility of short day to long day transition in *A. thaliana* for the *GIGANTEA* gene.**

Individual seedling traces from the data in Fig. 3(e), without normalisation to the mean, showing the consistency of regulation among seedlings (*n*=16 traces shown). Variation in the mean level of luminescence includes effects of seedling size and angle of leaves to the camera. White (day) and grey (night) shading represents the photoperiod.

**Fig. S6 Loop flexibility confers phase flexibility.**

The number of phases that are flexible under photoperiod variations for each of the models shown in Fig. 5. Here, the phase φ of a clock component (a peak or trough in expression level) is considered flexible if the corresponding dusk sensitivity ∂φ/∂*T_D_* is bounded away from 0 (dawn-locking) or 1 (dusk-locking).

**Fig. S7 Singular value spectra for the entrained clock models.**

The plots shows log_10_(σ*_k_*) for the singular values σ*_k_* of the entrained clock models considered in Fig. 4. The dotted line indicates the threshold ε used to calculate the flexibility dimension *d* in each case; this is defined as the number of σ*_k_*s with σ*_k_*>ε (Rand *et al*., 2004; Rand *et al*., 2006; Rand, 2008).
